# Changes in physical activity during COVID‐19 pandemic among Saudi Arabians: Results from a cross‐sectional study

**DOI:** 10.1002/hsr2.822

**Published:** 2022-09-12

**Authors:** Hassan Kobeissi, Abdelrahman M. Attia, Tasnim Atef Elgazzar, Jaffer Shah, Abubakr Bajaber, Sami Almustanyir, Ruaa Alsaeed, Razan Omer Khalifa, Ahmed Y. Azzam, Samar Hafida, Sherief Ghozy, Sheikh Mohammed Shariful Islam

**Affiliations:** ^1^ College of Medicine Central Michigan University Mount Pleasant Michigan USA; ^2^ Faculty of Medicine Cairo University Cairo Egypt; ^3^ Alfaisal University College of Medicine Riyadh Saudi Arabia; ^4^ New York State Department of Health New York USA; ^5^ Department of Internal Medicine Ministry of Health Riyadh Saudi Arabia; ^6^ October 6 University 6th of October City Egypt; ^7^ Beth Israel Deaconess Medical Center Harvard Medical School Boston Massachusetts USA; ^8^ Department of Neuroradiology Mayo Clinic Rochester Minnesota USA; ^9^ Institute for Physical Activity and Nutrition (IPAN) Deakin University Melbourne Victoria Australia

**Keywords:** COVID‐19, physical activity, Saudi Arabia, stroke

## Abstract

**Background and Aims:**

The COVID‐19 pandemic and the resultant change in sedentary behaviors have had immense health, economic, and social implications globally. As governments worldwide imposed lockdowns and curfews, the amount of time spent indoors greatly increased. This lead to a dramatic change in physical activity (PA) levels and profound consequences on daily routines. Our study aimed to investigate patterns of PA during the COVID‐19 pandemic among adults residing in Saudi Arabia.

**Methods:**

This cross‐sectional survey‐based study aimed to investigate patterns of PA during the COVID‐19 pandemic among adults residing in Saudi Arabia. The International Physical Activity Questionnaire was utilized to measure participants' PA levels between April 2021 and May 2021. Participants were then classified into three groups according to their PA level, and their PA levels and sedentary behaviors were analyzed.

**Results:**

We surveyed 463 participants, 315 (68%) of which were female and 134 (32%) of which were male with a median age of 23 (interquartile range, 21–35) years. Moderate‐to‐high PA was reported by 257 (55.7%) of the participants. There was a significant decrease in PA during the COVID‐19 pandemic and resultant lockdowns among the participants (*p* = 0.04), with higher rates of sedentary behavior among males than females (*p* = 0.14).

**Conclusions:**

The decline in PA is a profound challenge of the COVID‐19 pandemic that needs to be addressed by health practitioners and policymakers. Our study highlights the decline in PA levels seen during the COVID‐19 pandemic and the importance of promotional programs and interventions to increase PA among the Saudi Arabian population without compromising the essential health restrictions and social distancing.

## 1 INTRODUCTION

By the end of 2019, the world faced novel coronavirus strain named “COVID‐19.” The chief symptoms of this respiratory disease are fever, cough, and fatigue.^1^ Due to COVID‐19's high contagiousness, rapid spread, and lack of treatment options, governments worldwide imposed strict lockdowns to protect highly vulnerable populations, such as those with underlying health issues and comorbidities.^2^ In this context, Saudi authorities enforced specific measures to counteract the pandemic, including face masking, social distancing, distanced learning, border closures, and curfews.^3^ Such lockdown measures and stay‐at‐home directives increased pandemic‐related social isolation and fear.^4^ According to a local study, fear of COVID‐19 is variable based on sociodemographic characteristics and is associated with a cluster of negative lifestyle behaviors, such as increased food intake, decreased socialization, and increased health anxiety.^5^, ^6^, ^7^ Additionally, there was an increase in sleep disturbances during the COVID‐19 pandemic.^8^ These negative lifestyle behaviors are associated with a higher risk of developing medical complications and an increased mortality rate.^9^, ^10^, ^11^

Although these cautionary measures did mitigate the spread of COVID‐19, they also encouraged a shift toward sedentary lifestyles due to the closure of exercise facilities, work‐from‐home routines, and several other factors.^12^ As a result, there has been a noticeable increase in obesity rates due to the lockdown‐associated sedentary behaviors.^12^ Additionally, it has been documented that a lack of physical activity (PA) can reduce the effectiveness of the immune system as well as the vaccines' protection.^13^ Furthermore, it is widely accepted that physical inactivity, sedentary lifestyles, and obesity are risk factors for many health conditions, especially cardiovascular disease.^14^, ^15^ In fact, people who report sitting almost all of the time have a 54% higher risk of dying from all‐cause mortality or cardiovascular disease compared to those who report sitting almost none of the time.^16^ The current evidence shows that the pandemic has negatively affected the majority of individual lifestyles, while only a few could improve their daily habits by devoting more time to cooking healthy meals and at‐home workouts.^17^

Lifestyle behaviors are dynamic and can be influenced across different populations and different timings. Thus, the need for periodic assessment of lifestyle behaviors is evident to identify changes and act accordingly. Herein, we aimed to assess the PA levels of the Saudi population during the COVID‐19 pandemic and to evaluate the possible effects of sedentary behaviors on the general population's health status. Our study adds to the current literature by providing data from Saudi Arabia, a country in a region from which there has been limited data regarding lifestyle and PA trends during the COVID‐19 pandemic.

## 2 MATERIALS AND METHODS

### 2.1 Study design and population

This is a cross‐sectional online survey done through the widely used google forms. Participants aged ≥18 years and able to respond to an online questionnaire in English were invited to participate. Participants who partially completed the questionnaire and took <1 min to complete the survey were excluded from the analyses to avoid information bias. Results from the questionnaire were collected between April 2021 and May 2021.

### 2.2 Ethics

Ethics approval was obtained from the Human Research Ethics Committee (HREC) at King Fahad Medical City (IRB registration number H‐01‐R‐012). Data were collected anonymously. Study participants had the freedom to decline to answer any question and to withdraw from participation at any time.

### 2.3 Sampling

All participants fulfilling the inclusion criteria were invited to participate. The sample size was calculated according to the specific country setting for this global study. Snowball sampling was used to select the study participants.

### 2.4 Data collection

We used the structured survey questionnaire International Physical Activity Questionnaire (IPAQ). The survey was previously validated and translated into more than 10 languages.^18^ A screening question about the age of the participant was initially presented to confirm the participant's eligibility. Then, data collected included sociodemographics: age, gender, location of residence, marital status, living condition (alone or with families), the highest level of education, country of birth, profession; primary occupation, the impact of COVID‐19 on occupation; self‐reported comorbidities: hypertension, cardiovascular diseases, chronic respiratory diseases, diabetes, and cancer. Behavioral risk factors surveyed for included smoking status, alcohol intake. Health service utilization (in the last 4 weeks) was surveyed for, and was defined as consultation with a healthcare provider for any symptom, admission to the hospital including reasons for admission. Finally, exposure and contact of COVID‐19 were surveyed for, as defined as test and diagnosis of COVID‐19, close contact, isolation, and quarantine status and sedentary behavior measured by the global sedentary behavior questionnaire.

### 2.5 Statistical analysis

Data from the IPAQ questionnaire were analyzed based on the IPAQ recommendations for scoring protocol. Participants were classified into three different groups of PA considering the metabolic equivalent (MET)–min/week of the sum of walking, moderate‐intensity physical activities, and vigorous‐intensity physical activities: low active (<600 MET–min/week); moderate active (600 MET–min/week) and high active (3000 MET–min/week) (http://www.ipaq.ki.sese).

Continuous variables were expressed as median and interquartile range (IQR) because data were not normally distributed, while the categorical variables were presented as numbers and percentages. To compare different variables, we used the Kruskal–Wallis test for continuous variables and *χ*² exact test for categorical variables as appropriate. Multinomial logistic regression models were performed to determine the association between the predictor and dependent variables. The dependent variable (levels/categories of PA) was classified into three groups: low (reference category), moderate, and high. Hence, the multinomial regression model compared the probability in low versus moderate and low versus high categories. Univariate logistic regression was used to test the independent variables related to decreased PA during the COVID‐19 pandemic. Results were adjusted for age. A *p*‐value of 0.05 or less was considered statistically significant. Statistical analyses were performed using SPSS® software (version 26.0,) (IBM Corporation).

## 3 RESULTS

A total of 463 subjects were assessed in this study, whose ages ranged from 21 to 35 years with a median of 23 years (IQR, 21–35). Three hundred and fifteen (68%) of study participants were females. Two hundred and five (44.3%) of the enrolled participants engaged in a low‐PA, 182 (39.3%) in moderate‐level activity, and 76 (16.4%) in high‐level activity. Table 1 shows the characteristics of the study population.

**Table 1:** The characteristics of the study group

Participant characteristics	*n*	%
*Participant sociodemographics*		
Sex		
Female	315	68%
Male	134	30.9%
Age (median [IQR])	23 (21–35)	21–35
*Education level*		
Primary school	1	0.2%
Pre‐secondary school (intermediate phase)	12	2.6%
Secondary school	133	28.7%
University degree (Bachelor's or higher)	314	67.8%
Infected with COVID‐19	78	16.8%
*Participant PA*		
Time spent sitting (minutes per day) (median [IQR])	120 (20–360)	‐
PA levels		
Low	205	44.3%
Moderate	182	39.3%
High	76	16.4%
Decreased PA during COVID‐19 pandemic	271	58.5%
*Participant comorbidities*		
Had diabetes	17	3.7%
Had HTN	30	6.5%
Had stroke	1	0.2%
Had pulmonary embolism	3	0.6%
Had clots in any of the upper or lower limbs	6	1.3%
Had heart failure	5	1.1%
Had coronary heart diseases	4	0.9%
Had morbid obesity	46	9.9%

*Note*: Data are median (IQR) or *n* (%). *n* (%) unless otherwise stated (*n* = 463).

Abbreviations: HTN, hypertension; IQR, interquartile range; PA, physical activity.

**Figure 1:**
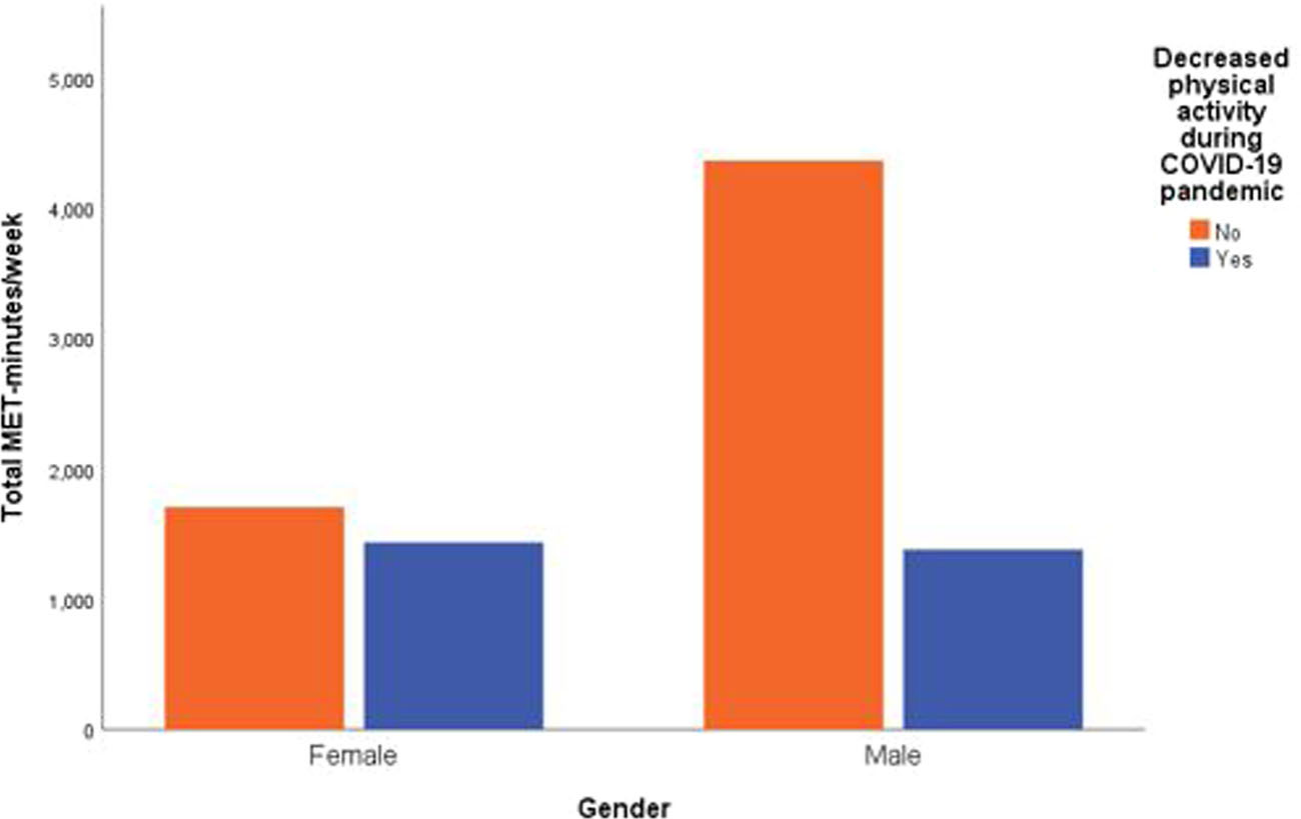
Physical activity during COVID‐19 pandemic stratified by gender

Table 2 demonstrates differences in PA with respect to the different studied variables. Male respondents were found to more frequently engage in high activity levels (*n* = 31, 21.7%) than women (*n* = 45, 14.3%). A noticeable reduction in male participants' PA level during the COVID‐19 pandemic was found. As demonstrated in Figure 1, males have higher total MET‐min/week than females with no reduction in PA.

**Table 2:** Differences in physical activity with respect to characteristics

Participant characteristics	Low (*n* = 205)	Moderate (*n* = 182)	High (*n* = 76)	*p*‐value
*Gender*				
Female	142 (45.1%)	128 (40.6%)	45 (14.3%)	0.14
Male	60 (42%)	52 (36.4%)	31 (21.7%)	
Age (median [IQR])	23 (21–35)	23 (21–33.5)	23.5 (21–38)	0.76
*Education level*				0.055
Primary school	1 (100%)	0	0	
Pre‐secondary school (intermediate phase)	5 (41.7%)	6 (50%)	1 (8.3%)	
Secondary school	55 (41.4%)	65 (48.9%)	13 (9.8%)	
University degree (Bachelor's or higher)	141 (44.9%)	111 (35.4%)	62 (19.7%)	
*Infected with COVID‐19*				0.97
Yes	35 (44.9%)	30 (38.5%)	13 (16.7%)	
No	167 (43.7%)	152 (39.8%)	63 (16.5%)	
*Had diabetes*				
Yes	4 (23.5%)	9 (52.9%)	4 (23.5%)	0.22
No	198 (44.7%)	173 (39.1%)	72 (16.3%)	
*Had HTN*				
Yes	13 (43.3%)	15 (50%)	2 (6.7%)	0.25
No	189 (44%)	167 (38.8%)	74 (17.2%)	
*Had stroke*				
Yes	1 (100%)	0	0	0.52
No	201 (43.8%)	182 (39.7%)	76 (16.6%)	
*Had pulmonary embolism*				
Yes	2 (66.7%)	1 (33.3%)	0	0.61
No	164 (42.3%)	158 (40.7%)	66 (17%)	
*Had clots in any of the upper or lower limbs*				
Yes	4 (66.7%)	2 (33.3%)	0	0.41
No	198 (43.6%)	180 (39.6%)	76 (16.7%)	
*Had heart failure*				
Yes	4 (80%)	1 (20%)	0	0.24
No	198 (43.5%)	181 (39.8%)	76 (16.7%)	
*Had coronary heart disease*				
Yes	4 (100%)	0	0	0.07
No	198 (43.4%)	182 (39.3%)	76 (16.7%)	
*Had morbid obesity*				
Yes	26 (56.5%)	14 (30.4%)	6 (13%)	0.19
No	176 (42.5%)	168 (40.6%)	70 (16.9%)	

*Note*: Data are median (IQR) or *n* (%). Categorical variables were analyzed using *χ*² test and the Kruskal–Wallis test for continuous variables. *p*‐value of 0.05 or less was considered statistically significant. *n* (%) unless otherwise stated (*n* = 463).

Abbreviations: HTN, hypertension; IQR, interquartile range.

Table 3 shows the multinomial logistic regression models that were performed to determine the association between the predictor variables and PA levels. Diabetes, hypertension, pulmonary embolism, clots in any of the upper or lower limbs, heart failure, and morbid obesity were associated with decreased PA during the COVID‐19 pandemic, but there was no significant relationship.

**Table 3:** Multinomial logistic regression models were performed to determine the association between the predictor variables and PA levels

	Unadjusted analysis of factors associated with PA levels				Adjusted analysis of factors associated with PA levels			
	Moderate‐level PA		High‐level PA		Moderate‐level PA		High‐level PA	
	OR (95% CI)	*p*‐value	OR (95% CI)	*p*‐value	OR (95% CI)	*p*‐value	OR (95% CI)	*p*‐value
*Sex*								
Female	1.040 (0.669–1.617)	0.861	0.613 (0.355–1.061)	0.08	1.1 (0.64–1.88)	0.727	0.49 (0.25–0.96)	0.039
Male	Ref		Ref		Ref		Ref	
Age	0.996 (0.978–1.014)	0.641	1.004 (0.982–1.027)	0.696	0.99 (0.97–1.01)	0.585	0.98 (0.95–1.01)	0.417
*Education level*								
Primary school	‐		‐		‐		‐	
Pre‐secondary school (intermediate phase)	1.524 (0.453–5.125)	0.496	0.455 (0.052–3.974)	0.476	1.39 (0.29–6.48)	0.675	0.52 (0.05–5.53)	0.59
Secondary school	1.501 (0.970–2.324)	0.068	0.538 (0.274–1.055)	0.071	1.1 (0.714–1.91)	0.536	0.53 (0.259–1.08)	0.082
University degree (Bachelor's or higher)	Ref		Ref		Ref		Ref	
*Infected with COVID‐19*								
No	1.062 (0.622–1.813)	0.826	1.016 (0.505–2.044)	0.965	1.1 (0.65–215)	0.571	0.84 (0.39–1.77)	0.654
Yes	Ref		Ref		Ref		Ref	
*Had diabetes*								
No	0.388 (0.118–1.283)	0.121	0.364 (0.089–1.492)	0.160	0.21 (0.04–1.06)	0.060	0.188 (0.02–1.29)	0.089
Yes	Ref		Ref		Ref		Ref	
*Had HTN*								
No	0.766 (0.354–1.656)	0.498	2.545 (0.561–11.552)	0.226	0.56 (0.20–1.54)	0.265	2.89 (0.49–16.93)	0.23
Yes	Ref		Ref		Ref		Ref	
*Had pulmonary embolism*								
No	1.927 (0.173–21.461)	0.594	‐	‐	0.60 (0.02–13.36)	0.747	‐	‐
Yes	Ref		‐		‐		‐	
*Had clots in any of the upper or lower limbs*								
No	1.818 (0.329–10.046)	0.493	‐	‐	1.005 (0.14–7.11)	0.996	‐	‐
Yes	Ref		‐		Ref		‐	
*Had heart failure*								
No	3.657 (0.405–33.019)	0.248	‐	‐	10.26 (0.47–222.79)	0.138	‐	‐
Yes	Ref		‐		‐		‐	
*Had morbid obesity*								
No	1.773 (0.895–3.511)	0.101	1.723 (0.680–4.368)	0.251	1.73 (0.74–4.01)	0.200	0.87 (0.30–2.51)	0.809
Yes	Ref		Ref		Ref		Ref	

*Note*: *p*‐value of 0.05 or less was considered statistically significant. *n* (%) unless otherwise stated (*n* = 463).

Abbreviations: HTN, hypertension; PA, physical activity.

## 4 DISCUSSION

The main aim of this study was to examine the effects that COVID‐19 lockdowns had on the PA levels among Saudi adults. Our results indicate that only 55.7% of respondents reported moderate‐to‐high PA levels during the pandemic. This is a cause for concern as the WHO's Global Recommendations on Physical Activity for Health mentions that low levels of physical inactivity are risk factors for several conditions that can worsen COVID‐19 outcomes, such as hypertension, diabetes, and cardiovascular diseases.^19^ Furthermore, with a slight reduction in PA, effects can compound over time as the pandemic and lockdowns could last for months to years.

The secondary aim of this study was to investigate the relationship between different adverse health conditions, such as diabetes, hypertension, heart failure, and so on, and the amount of PA reported by patients. Interestingly, we did not find an association between PA groups and comorbidities. There are several possible causes of this, two of which are that either the decreased activity levels have no effect on the incidence of these diseases, or these patients were not taking the necessary measures to stay home safely. We also suspect that this may be due to a power issue since only a small proportion of respondents had comorbidities. For example, only five (1.1%) respondents had congestive heart failure, while four (0.9%) had coronary heart disease. With a larger sample size, we might see an association between PA levels and different comorbidities, as it has been reported that patients with comorbidities, such as diabetes, tend to partake in lower levels of PA.^20^ However, our study reported a trend toward a decrease in PA among all groups during the COVID‐19 pandemic. Hence, our findings show a decrease in PA levels across populations due to the lockdown measures to mitigate the spread of COVID‐19.^21^

Our findings also highlight the importance of promotional campaigns and interventions to raise awareness and increase PA among adults in Saudi Arabia. The low physical activities among residents of Saudi Arabia, especially females, were highlighted in a previous survey which also showed that females were significantly less active.^22^ Educating the Saudi community about the importance of PA and understanding the challenges of implementing nationwide promotional programs, including the COVID‐19 pandemic restrictions and social barriers, is essential before developing action plans.

Our study had several limitations. First of all, we faced a possible recall bias as our data was based on participants' memory and was self‐reported. Second, the study design was cross‐sectional, meaning there may have been a nonresponse bias.^23^ As such, we might expect that participants whose habits did not change during the pandemic could be less inclined to complete the form. Our study also faced some study‐design‐specific limitations. In particular, only participants with internet access could access the questionnaire, the questionnaire was administered in English, and the survey was self‐administered. Furthermore, it is important to consider that not all papers that have been published in our area of interest are qualitatively well‐performed papers.^24^

## 5 CONCLUSION

In conclusion, this study demonstrated that a significant decrease in PA occurred during the COVID‐19 pandemic and resultant lockdowns among the general population of Saudi Arabia. The prevalence of physical inactivity may lead to further health complications if reinforced and sustained in the long term. These findings may contribute to providing evidence‐based recommendations for healthcare professionals and policymakers. The long‐term effects of this decline in PA could pose a wide‐scale public health problem in the future, and these trends should be taken into consideration in the event of a new pandemic that requires isolation. Health awareness and behavioral intervention are necessary to overcome the profound challenges of the COVID‐19 pandemic and to help reverse the trend of decreased activity that occured.

## AUTHOR CONTRIBUTIONS

**Hassan Kobeissi**: Conceptualization; Data curation; Investigation; Writing–original draft; Writing–review & editing. **Abdelrahman M. Attia**: Formal analysis; Writing–original draft; Writing–review & editing. **Tasnim Atef Elgazzar**: Methodology; Writing–original draft; Writing–review & editing. **Jaffer Shah**: Formal analysis; Writing–original draft. **Abubakr Bajaber**: Validation; Writing–review & editing. **Sami Almustanyir**: Investigation; Resources; Software; Writing–review & editing. **Ruaa Alsaeed**: Project administration; Software; Visualization. **Razan Omer Khalifa**: Investigation; Project administration; Writing–original draft; Writing–review & editing. **Ahmed Y. Azzam**: Investigation; Validation; Writing–review & editing. **Samar Hafida**: Conceptualization; Investigation; Software; Supervision; Validation; Writing–review & editing. **Sherief Ghozy**: Conceptualization; Formal analysis; Investigation; Methodology; Project administration; Resources; Supervision; Validation; Visualization; Writing–review & editing. **Sheikh Mohammed Shariful Islam**: Project administration; Supervision.

## CONFLICT OF INTEREST

The authors declare no conflict of interest.

## ETHICS STATEMENT

Ethics approval was obtained from the Human Research Ethics Committee (HREC) at King Fahad Medical City (IRB registration number H‐01‐R‐012).

## TRANSPARENCY STATEMENT

The lead author Jaffer Shah affirms that this manuscript is an honest, accurate, and transparent account of the study being reported; that no important aspects of the study have been omitted; and that any discrepancies from the study as planned (and, if relevant, registered) have been explained.

## Data Availability

The data that support the findings of this study are available from the corresponding author, Hassan Kobeissi, upon reasonable request.
